# Photo-voicing experiences of teenage mothers with teenage pregnancy and motherhood in Western Uganda

**DOI:** 10.1371/journal.pone.0335413

**Published:** 2025-11-13

**Authors:** Linda Grace Alanyo, Enos Mirembe Masereka, Pebalo Francis Pebolo, Emmanuel Kimera

**Affiliations:** 1 Department of Nursing and Midwifery, Mountains of the Moon University, Fort Portal, Uganda; 2 Department of Sexual and Reproductive Health, Gulu University, Gulu, Uganda; 3 Department of Public Health, Mountains of the Moon University, Fort Portal, Uganda; PLOS: Public Library of Science, UNITED KINGDOM OF GREAT BRITAIN AND NORTHERN IRELAND

## Abstract

Teenage Pregnancy and Motherhood (TPM) pose significant global public health challenges, particularly in Sub-Saharan Africa with Uganda being among the countries most affected. In this phenomenological study we aimed to understand the lived experiences of teenage mothers regarding TPM, and to develop advocacy materials and methods to combat TPM. We recruited 14 teenage mothers, aged 16–19 years, who were receiving care at a high-volume hospital in Fort Portal City, Western Uganda. Photovoice, a participatory action research method was used, involving five group sessions. Participants documented their TPM experiences through photographs and narratives, followed by group discussions. Data were analyzed using phenomenological hermeneutics, with themes emerging from participants’ lived experiences. Teenage pregnancy and motherhood were understood to be unexpected and undesirable by the girls who experienced them. These situations were often marked by attempts to conceal the pregnancy, such as fleeing from home, and were compounded by insufficient material and psychosocial support. The girls also faced conflicting emotions about having children, challenges related to childbirth and childcaring as well as strained relationships with their families or partners. The unexpected, undesired, and challenging nature of TPM for this population highlights the need for comprehensive societal and systemic interventions to prevent TPM and to provide material and psychosocial support to those that find themselves in this situation. This can be through sexuality education to teenage girls and economic support for teenage mothers. Incorporating teenage mothers’ lived experiences into advocacy efforts offers a novel approach to addressing the TPM challenge in the setting of western Uganda. Teenage pregnancy is a deeply stigmatized and challenging experience for young girls, often leading to social isolation, concealment, and insufficient support, highlighting the urgent need for comprehensive societal and systemic interventions.

## Introduction

Teenage Pregnancy and Motherhood (TPM) present significant global challenges, both in terms of public health and societal impact [1]. The burden is disproportionally high in Sub-Saharan Africa (SSA), accounting for over 95% of the cases [2]. Uganda, specifically, stands out with one of the highest rates of TPM in SSA, where 25% of women become pregnant before the age of 18 [3]. Given that 77% of Uganda’s population is below 25 years, this translates into a significant number of girls involved in TPM. While the teenage period is a critical stage that ushers one into adulthood, TPM is a major hinderance for many girls to achieve a smooth transition [4]. Each case of TPM in Uganda presents with unique drivers and experiences, necessitating an in-depth understanding of the phenomenon.

Extant literature highlights various factors contributing to the high incidence of teenage pregnancies. These factors include individual behaviors, peer pressure, socio-cultural influences, and structural issues [5,6]. In Uganda, the implementation of the national comprehensive sexuality education has been constrained by conservative cultural and religious attitudes and norms around women’s sexuality and unequal power relations [7]. Consequentially, many teenage girls lack awareness about sexual and reproductive rights and responsibilities and are prone to misinformation [8]. Many, easily yield to peer pressure that emanates from their social circles [9]. Additionally, the rise of internet and social media access has complicated matters, exposing teenagers to uncensored sexual content [10]. The African traditional guidance from elders, once a cornerstone in navigating matters of sex and sexuality, has diminished, replaced by commercial influences promoting reckless sexual behavior [11]. Also, cultural norms and practices that condone early marriage and early childbearing [12] as well as gender inequalities and power imbalances, including sexual coercion [13] contribute significantly to TPM. Furthermore, poverty and socioeconomic inequalities, which limit educational and economic opportunities for young women expose teenage girls to sexual exploitation and lead to TPM [14].

The journey of TPM is fraught with numerous biomedical and social challenges, distinct from those experienced by older mothers [15,16]. TPM can profoundly shape the lives of young girls, impacting their physical, emotional, and socio-economic wellbeing. Often unplanned and unwanted [17], these pregnancies contribute to a range of complications for both mother and child, including obstructed labor, obstetric fistulas, preterm birth, and low birth weight babies [18–20]. Teenage mothers also face increased risks of unsafe abortions, sexually transmitted infections, and sexual violence, compounded by limited access to medical services [21]. Moreover, societal stigma surrounding TPM exacerbates the psychological burden on pregnant teenagers, teenage mothers, and their immediate associates (family, peers, and partners) [22,23]. This stigma may lead to feelings of shame, isolation, and psychological distress, impacting the mental health and well-being of teenage mothers. TPM disrupts girls’ education, leading to higher dropout rates and reduced opportunities for future employment and economic independence [24], consequently relegating teenage mothers to the fringes of society.

The complex interplay of drivers and negative sequels of TPM in Uganda requires the involvement of the entire socio-ecology around teenage girls for a comprehensive and durable solution. At the macro-policy level, the Ugandan government implements a universal primary and secondary education policy [25] that reduces the fiscal burden of attaining primary and secondary education to ensure longer stay in school. Vocational training is also available through the ‘skilling Uganda’ program [26] for those teenagers who find academic work challenging. A favorable policy-legal environment is also available to, ideally, protect the rights of teenage girls and to address the underlying socio-cultural drivers of teenage pregnancy, such as child marriage and gender inequality [27]. Furthermore, youth-friendly Sexual and Reproductive Health (SRH) services [28,29] are offered at many public healthcare facilities to encourage young people to seek SRH services within a safe space setting. However, teenage girls still fall through these safety nets and get pregnant.

TPM is a well-researched topic, with numerous studies focusing on its drivers, health implications, and socio-economic outcomes for teenage mothers. However, limited research focuses on exploring TPM through the lived experiences of teenage girls, particularly using advocacy and empowerment methods like photovoice. Photovoice is a powerful tool for capturing personal experiences, analyzing community concerns, and developing solutions to challenges faced by marginalized groups, such as teenage mothers. By leveraging the experiences of teenage mothers through this method, we offer a novel approach to address TPM. Additionally, through the advocacy, inherent in photovoice, we aim to empower school-going teenagers with knowledge to combat TPM through an effective peer-to-peer approach [30]. This endeavor aligns with broader Sustainable Development Goals (SDGs) [31] of promoting health and well-being (SDG3), education for all (SDG4), and gender equality (SDG5). Our objectives were twofold: 1) to understand the experiences of teenage mothers regarding TPM and 2) to generate materials and methods for advocacy against TPM.

## Methods

### Study design, settings, and participants

We conducted a phenomenological study, based on the philosophy of Maxi van Manen [32], with a group of 14 teenage mothers (16–19 years) selected purposively from a high-volume hospital in Fort Portal city, western Uganda. Teenage mothers were recruited at the immunization clinic after receiving care. Participant selection was based on the inclusion criteria of: being a teenage mother (with a live baby less than one year), willing to participate in 5 group sessions, and being able to speak the local language (Rutooro). Exclusion applied to those potential participants whose babies were sick and those who came from outside Fort Portal city. During the selection, healthcare workers referred potential participants to the research team members who were on standby at a private waiting area near the immunization clinic. Efforts were made to include participants of varied ages as much as possible. Following informed written consent, each participant was invited to participate in 5 group sessions over the course of 2 months (4^th^ June to 30^th^ July, 2024) at a public meeting facility near the hospital.

### Procedure

Photovoice, a participatory action research method [33] was used to elicit and make meaning of teenage mothers’ experiences. We followed pragmatic steps by Kimera and Vindevogel suitable for young, marginalized participants in resource limited settings [34].

In the first session, participants were introduced to the photovoice concept and procedures. We explained the goal and purpose of the project, discussed the ethics of photography as well as appropriate and inappropriate photos. The second session involved challenging participants to remember their pregnancy, childbirth, and mothering experiences, think through them critically, visualize and present them as hand drawn pictures in notebooks that were provided. Although highly amateur, these pictures set stage for the conceptualizations of project photographs. In the third session, we gave each participant a camera (previously used in another study), taught them basics of using a digital camera, and we supervised pilot photography around the meeting venue. The research team took turns to support participants by carrying their babies and practically showing them how to take good photos. Participants were then assigned to take photos to show their experiences with Teenage Pregnancy and Motherhood (TPM) in their living situations over a period of 2 weeks. For each project photo, they were instructed to write a corresponding brief narrative (for those that could write) in their notebooks. It was also made clear to them that they were free to take their own photos as motivation for their involvement in the project. In the fourth session, photographs were obtained from the cameras of participants and stored in the computer of the research team within folders marked with participant assigned numbers (Teen 1 to Teen 14). Each participant selected photos to discuss in a group and those for their own albums. The fifth session was a group discussion of the photos, moderated by the research assistant (native speaker of the local language) and using a variation of the SHOWeD technique, a well-established technique to guide photovoice discussions [35]: What do you See here? What is Happening? How does this relate to Our lives (How does this photo show your experience with TPM?) Why does this problem exist? What can we Do about it? The discussions were conducted in a private quiet room, and they were audio recorded with consent from participants. In turns, participants displayed their photos using a laptop and projector provided by the research team.

### Actionable strategies against TPM

To address the project’s second objective, participants were asked to suggest strategies for reaching teenage girls with information to help them avoid pregnancy and to engage local policymakers in advocating for the social welfare of teenage mothers. All participants endorsed the idea of organizing photo-text exhibitions in secondary schools within the study area, as most teenagers are likely to be in school. One participant stated that: *“Me I think this information needs to reach all girls like those who are still schooling. You see, before it happens to you, you may think it does not exist. They need to see these photos and get some lessons from them”* (Teen 10, 17-year-old school dropout). These exhibitions were planned and successfully conducted, though participants opted not to take an active role in them. Additionally, the idea of hosting an annual youth conference to present the study’s findings was proposed by participants, and the research team considered this as a potential platform for sharing the results.

*“Here in [name of place], they organize a conference for young people every year to tell them AIDS and other things that young people face. It can be good to use that conference to share information with young girls and boys so that they avoid these problems”* (Teen 6, a 17-year-old school dropout)*.*

### Data management and analysis

The initial analysis began by identifying potential themes to be captured in the photos in collaboration with the participants. This process took place during a reflective session where participants were guided to conceptualize and represent their experiences with TPM through hand-drawn pictures.

The data for further analysis included audio files, field notes, and photographs with accompanying captions. The audio files were transcribed verbatim, and the transcripts, along with the photo captions, were translated into English by a postgraduate student who was a native speaker of the local language and fluent in English. LGA then compared the translated transcripts with the originals to ensure translation accuracy. The transcripts were refined by removing redundant and repetitive words and phrases. Additionally, where participants used vague pronouns such as ‘it,’ ‘thing,’ ‘they,’ ‘he,’ and ‘she,’ we clarified these references by specifying the object, place, or person’s relationship in square brackets.

Data analysis was based on phenomenological hermeneutics, involving three researchers (LGA, EK, and EM). This method involves interpreting text to gain a deeper understanding of the phenomena through the stories of those who have lived it [36]. Our approach was informed by the philosophical underpinnings of Ricoeur [37] and Van Manen [38]. Following the hermeneutic circle [39], we repeatedly read the transcripts and listened to the audio files to immerse ourselves in the data, gaining an initial holistic understanding of the participants’ experiences. We documented our reflections in memos. By examining the photos and their captions, we continually reflected on how the various pieces of data related to our initial understanding, allowing us to refine our interpretations. Throughout this process, we contextualized participants’ experiences. To remain sensitive to the data and avoid misinterpretation, we consciously set aside our prior knowledge and assumptions about TPM. This was especially important for two authors, LGA and EM, who had previously provided clinical care to teenage mothers. Potential biases were further minimized through joint analysis that involved EK from another discipline.

In the final analysis, LGA, EM, and EK independently applied open codes to two transcripts. These codes were written on sticky notes and displayed on tables. In iteration, we reviewed the codes and revisited the coded data to reach a consensus on the open codes that represented participants’ discourses. We then grouped the open codes based on conceptual similarities and assigned more abstract interpretive category names to create overarching themes. We initially planned to develop separate themes for pregnancy, childbirth, and childcare, but this was dropped since it led to too much overlap. For example, the experience of lacking support was consistent throughout and thus would be repeated in all the categories. LGA completed the coding using the developed structure (Table 1) in Nvivo version 14 for easier data retrieval.

**Table 1:** Overarching themes and subthemes that emerged from the analysis.

Overarching themes	Subthemes
Being surprised and attempting to conceal the pregnancy	Wondering what to do
	Being confused and surprised
	Getting first signs of pregnancy
	Running away from home
	Fearing imprisonment for partner
	Revealing pregnancy to relatives first
	Fearing parental reaction
Living with ambivalence and strained relationships	Happy to get a baby
	Parents being disappointed
	Parents happy to see baby
	Regretting having given birth
	Mixed reactions from community members
	Mistreatment from partner
	Abandonment by partner
Receiving insufficient support	Attending antenatal care
	Partner’s role in care and support
	Not getting good care at health facility
	Lacking money
	Relatives paying hospital bills
Living through predicaments and diminished social value	Baby crying too much
	Being under-looked
	Baby falling sick all the time
	Made to do adult roles
	Experiencing abortion
	Getting difficulties during delivery
	Getting post-delivery complications
	Experiencing near abortion
	Getting much pain during labor

### Ethics

This study was part of a larger research project with a goal of addressing sexual and reproductive challenges for adolescent girls in western Uganda. Ethical clearance for the project was obtained from the research and ethics committee of Mbarara University of Science and Technology (MUST-2023-1266). Administrative clearance was obtained from the hospital director and the in-charge of the immunization clinic. We obtained informed written consent from all participants at the time of recruitment. Those below 18 years were considered emancipated minors since they were already mothers and either living independently or with their partners and thus, we did not seek consent from their parents/caretakers. All discussions were conducted in a private place and were moderated by a trained research assistant with over 5 years working experience with teenage girls. Each participant was reimbursed for transport after each session at a rate of 6.6 USD. Audio files, photos, and transcripts were anonymized with assigned numbers and secured by password in the computer of the research team.

## Findings

### Characteristics of participants

We included 14 teenage girls aged 16–19 years in the study. Of these, 10 (71%) had children aged 1 year or younger, while the remaining participants were pregnant. Except for one participant who had two children, all the others had their first baby or were pregnant for the first time. Twelve participants (86%) had been attending school prior to their pregnancies, but none had resumed their education since then (Table 2).

**Table 2:** Characteristics of participants.

Characteristic	Frequency	Percentage (%)
**Age (years)**		
16-17	9	65
18-19	5	35
**Mean age (SD)**	17.21(1.01)	
**Marital status**		
Married	2	14
Single	12	86
**Childbirth status**		
Has a child	10	71
Has a pregnancy	4	29
**Schooling status**		
Dropped out of school due to pregnancy	12	86
Dropped out of school before pregnancy	2	14

The four overarching themes from the analysis are presented below, in order of the extent to which they encompass the participants’ experiences.

#### Being surprised and attempting to conceal the pregnancy.

Except for a few instances of forced sex, most teenage girls ventured into sexual relationships voluntarily. However, their stories reveal that neither they nor their partners were prepared for pregnancy or the resulting childbirth, despite not using available contraceptives. The knowledge of their pregnancies came as a surprise, leading to anxiety and confusion about how to handle the situation, especially given their young age and unmarried status. This is typified in the quote below from Teen 7.

*“When I told my boyfriend, he accepted he didn’t refuse but he asked me what should we do? Because you and I are still at school. He asked me should we tell the parents? I tell my parents, and he tells his parents. I first told him it is not possible, he asked me why”* (Teen 7, 17-year-old, school dropout).

**Fig 1:**
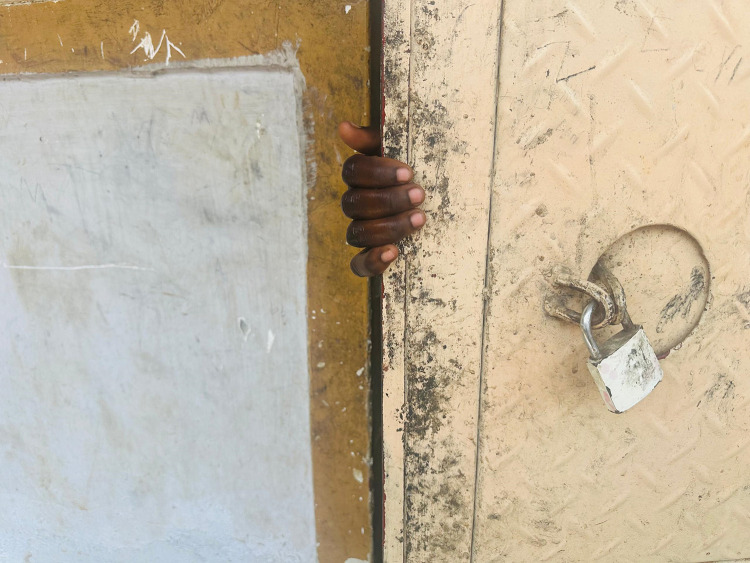
*“You may try to hide here and there but the pregnancy or the child will soon be seen, and people will get to know. It is just a matter of time”* (Teen 1, 18-year-old, non-schooling).

In all cases, teenage girls began to suspect they were pregnant when they missed their menstrual periods, and they attempted to hide the pregnancy. This concealment was driven by various fears, with the most significant being how their fathers would react upon discovering their pregnancy or the birth of a child. The threat of imprisonment for their partners was a likely response from fathers, especially when the involved teenagers were under 18 or still in school. To avoid this, all the girls sought ways to leave home, staying with other relatives (such as a brother, sister, or aunt) under the pretense of a short visit. However, this concealment was short-lived, as early physical signs of pregnancy began to show. These signs led their relatives to question them about the possibility of being pregnant. Realizing they could no longer hide the pregnancy caused many of the girls to become anxious and even cry. The photo by Teen 1 (Fig 1) portrays the struggles to hide what would eventually emerge.

“*When I reached there, after two weeks my sisters started seeing my life was not as usual, I had changed and looked like a pregnant woman. They [sisters] first feared to ask me because they [sisters] knew I was not easy. They [sisters] went to work and talked about it. When they [sisters] came back with my eldest brother asked me that are you pregnant? Thats because you have all signs of pregnancy”* (Teen 2, 16-year-old, school dropout).

Disclosing the pregnancy to parents was understood to involve an advocate, usually a brother, sister, or aunt, who would inform the mother, and the mother would then tell the father. In two cases, the girls shared that their fathers only learned of the pregnancy after the babies were born.

*“They [sister and brother] first called mummy at home she is the first one they told not dad, and they explained to her. Like a parent, she first got annoyed then later she calmed down. She asked them [sister and brother] to let me go back home, they [sister and brother] said no Daddy is a problem”* (Teen 11, 19-year-old school drop-out).

#### Living with ambivalence and strained relationships.

Participants’ stories and photos revealed mixed emotions regarding their pregnancy and childbirth experiences. These conflicting feelings were evident both within the teenage girls themselves, and among their parents and community members. While most teenage girls were initially unhappy about being pregnant, the arrival of their baby brought them some happiness. However, when faced with challenges of motherhood, such as a sick or constantly crying baby, some girls regretted having given birth. The girls expressed similar sentiments by parents, some of whom showed little interest in their grandchildren, as they had hoped their daughters would achieve academic success after investing in their education.

*“At that time, we looked for what to do after telling Mummy. So here Mummy told me this [pregnancy] is what you have paid me with after all the money I have put in you. We have been giving you everything you needed so these are the benefit you have brought for us”* (Teen 5, 19-year-old, non-schooling).

From the participants’ discourses, it became clear that community members who had sour relationships with the teenage girls or their families took satisfaction in learning that the girls had become pregnant or had given birth, believing that this event would negatively impact their future. Conversely, those who had good relationships with the girls expressed disappointment.

**Fig 2:**
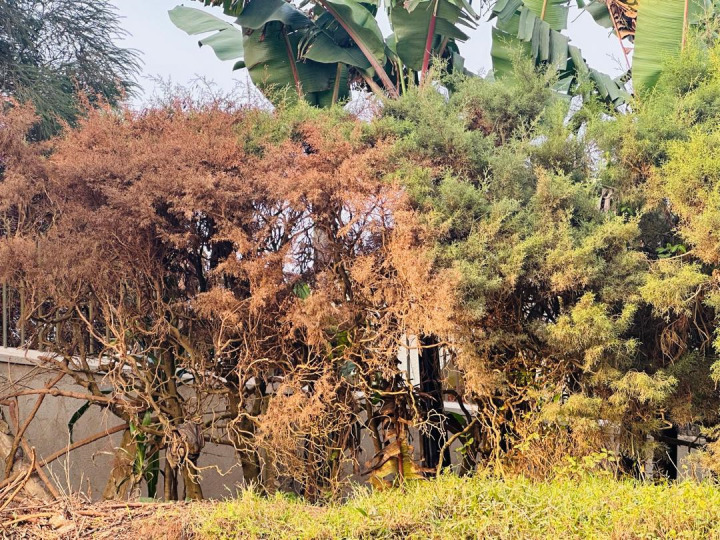
*“On one side you feel like it is okay to have a baby and you are happy and at another time you just feel like I wish I did not have this child when everything seems to be dying”* (Teen 10, 17-year-old, school dropout).

Our reflections on participant narratives sprout an understanding that these ambivalences strained relationships between teenage girls and their parents, partners, and community members. Partners who initially expressed willingness to provide care and support resorted to mistreatment and abandonment because they were not ready for the relationship and the baby. The photo from Teen 10 (Fig 2) illuminates this ambivalence.

#### Receiving insufficient support.

The teenage girls in this study were mostly unemployed and many were still in school when they became pregnant, making them dependent and unable to support themselves. The unexpected pregnancy added an emotional burden to the medical and material needs associated with pregnancy and childbirth. The strained relationships with significant people in their lives, as discussed in the previous theme, led to inadequate psychosocial, medical, and material support.

Although all the teenage girls reported attending antenatal care and delivering at healthcare facilities, many expressed dissatisfactions with the care and attention they received from healthcare workers. Their stories highlight a gap between their expectations and the reality of the care provided. For instance, some girls expected free services, but healthcare workers asked for payments. Another girl expected to receive immediate support upon arriving at the hospital to deliver her baby, but instead, she was told to sit and wait.

*“After sometime she [Healthcare worker] came back and saw me and bypassed me. I asked her you woman they have sent me here to do scan and they told me it is for free she said why do you like free things, free things are not here they got finished. If you want them go to town and become a beggar”* (Teen 11, 19-year-old school dropout).

**Fig 3:**
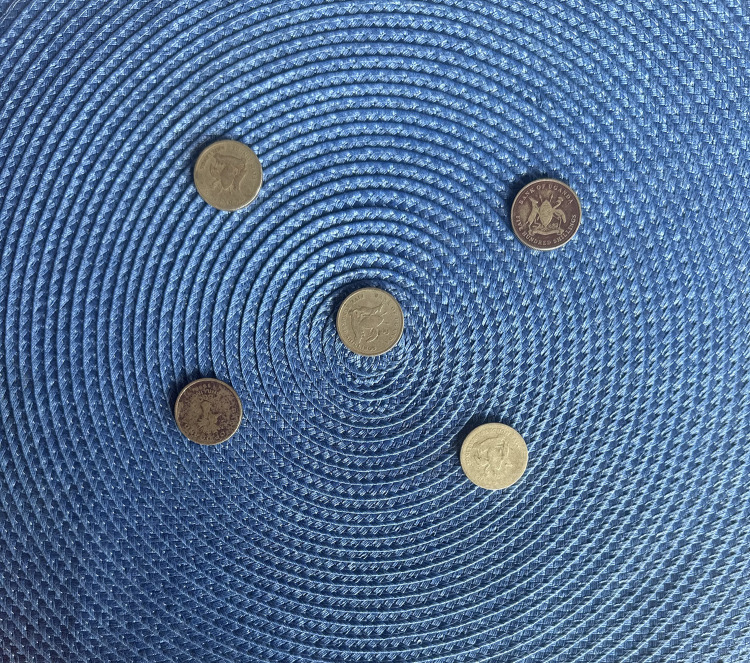
*“I used to dig in people’s shambas* [Gardens] *to get some money when I was pregnant but it was not even enough to take care of me”* (Teen 12, 18-year-old school drop out).

In a few cases, teenage girls received support from their partners, who had accepted responsibility for the pregnancy and baby, helping to cover medical bills and other expenses. However, for most, medical bills were paid by relatives, such as in-laws and aunts. A significant number of girls who did not receive support from their partners struggled to provide clothing and food for their babies. Teen 12 portrays lack of financial support from the people around her to navigate challenges of the pregnancy (Fig 3).

*“When I told the man that I was pregnant, he refused that it was not his pregnancy. I stayed with my pregnancy without any help from him. When labor started I went to* [name of healthcare facility] *I called him and told him they have asked for money I request for money he said I will not send you money because that pregnancy is not mine and that baby is not mine”* (Teen 9, 17-year-old non-schooling).

#### Living through predicaments and diminished social value.

**Fig 4:**
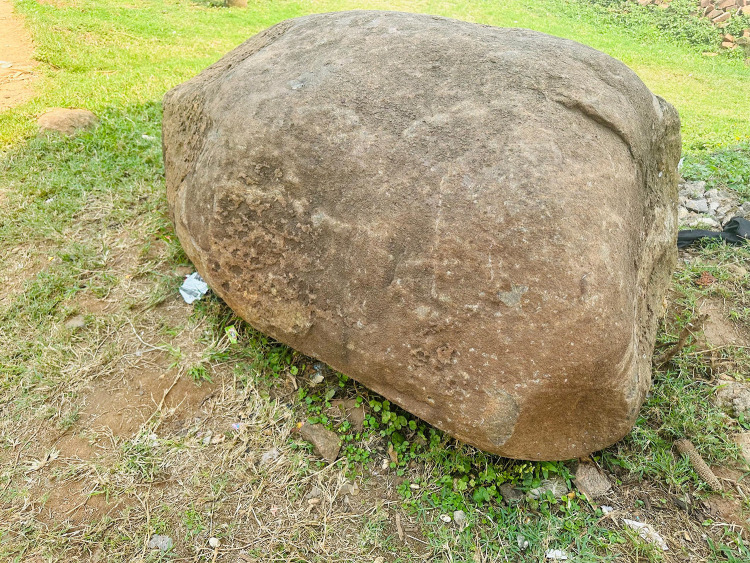
*“Sometimes life is as hard as this stone, everything you plan does not workout, the baby is disturbing, you don’t have money, and people are just looking at you like that”* (Teen 6, 17-year-old school dropout).

Participants described their journey from pregnancy to motherhood as fraught with unexpected challenges. Teen 6 captured a photo of a stone (Fig 4) to symbolize the hardships in her life. A 19-year-old girl shared that she had an abortion with her first pregnancy, while two other girls recounted how medical interventions had prevented imminent miscarriages. Many experienced their pregnancies with frequent illnesses and abnormal fetal positions.

All the teenage girls reported facing numerous ordeals during childbirth, including perineal tears, severe labor pains, excessive bleeding, and even a torn uterus.

During the child-caring period, they were distressed by their babies’ constant crying and frequent illnesses, which led to sleepless nights. This stress was compounded by the inadequate support mentioned in earlier themes. Furthermore, the girls felt that society viewed them as having reduced social value and being a burden, particularly those who lived with their parents.

“*At home they don’t see you as an important person. Because you have given birth from home, your children are disturbing them[relatives]”* (Teen 14, 17-year-old, school dropout).

Those staying with relatives were required to make financial contributions in the home since they had taken up adult roles [giving birth] despite being unemployed.

*“Some of us stay with our sisters. Sometimes our sisters leave everything on our heads like buying things to use at home. Remember we are not working and my husband the phone is off. So, you have to look around and get something some time we stay hungry. Even they reach an extent of hiding things like food just because you have not contributed”* (Teen 1, 18-year-old, non-schooling)*.*

## Discussion

In this phenomenological inquiry, we answered the question: what is it like to be a pregnant teenager and a teenage mother? Teenage pregnancy and motherhood (TPM) were understood as unexpected and unwelcome by this group of participants. The lives of these teenage girls were marked by efforts to conceal their pregnancies, insufficient support, mixed emotions, challenges related to pregnancy and childbirth, and strained relationships. To better understand their experiences, we situated our participants as young, inexperienced, first-time mothers who are still dependent on others. These girls are often negotiating their first sexual relationships while continuing their education or after dropping out of school due to financial difficulties in their households. Our goal was to explore the distinctiveness of their experiences in comparison to adult women within the local context of the study.

In contrast to some studies that reported positive reactions from teenage girls and their families upon discovering a pregnancy [40,41], our participants experienced it as unexpected and unwanted. This is consistent with previous research that identified teenage pregnancy as a largely unplanned and unwanted event in many low-resource settings [42,43]. The efforts to conceal the pregnancy, driven by fear of parental reactions and societal judgment, underscore the stigma associated with teenage pregnancies in this contex. This stigma is deeply rooted in cultural norms that emphasize marriage and education before childbearing [44], making it difficult for teenage mothers to seek and receive the support they need. In the local context of this study, and more broadly in Uganda, a pregnancy occurring outside of marriage is considered a dishonor and a taboo for the girl’s parents [45]. Additionally, with the legal age of consent being 18 years [46], men who impregnate girls under the age of 18 years may face criminal charges. Teen mothers are thus stigmatized for violating age and cultural norms. This explains why many girls attempted to conceal their pregnancies and even eloped with their partners, who were often unprepared for a serious relationship. Despite the socio-cultural and legal safeguards, TPM continues to manifest, suggesting the need for alternative strategies such as making contraceptives accessible to teenage girls. The experience of TPM being unwanted and unexpected makes clear the unmet need for contraception among this group. However, promoting contraceptive use among teenagers has evoked controversy in Uganda, with realists advocating for it as a practical solution, while moralists argue that it could encourage sexual immorality among young girls [47]. Except for one girl who reported being raped, our findings indicate that the girls willingly engaged in unprotected sex. This presents a significant challenge not only in terms of unintended pregnancies but also in controlling HIV, in a country with a high prevalence of the infection in the general population. The findings also align with existing evidence showing a high incidence of HIV among adolescents [48]. Thus, a dual approach that addresses both teenage pregnancies and HIV prevention is essential. To achieve this, promoting condom use among sexually active teenagers is crucial. It is also important to explore the barriers to accessing and using condoms within this age group to understand what hinders this effective intervention against both HIV and unintended pregnancies.

Consistent with findings from similar studies [49], our research highlights several medical and health challenges faced by teenage mothers from pregnancy through childbirth and the postpartum period. Significant issues included abortions, excessive pain during labor, improper fetal presentation, perineal lacerations, and infections. Compared to adult primigravida, young mothers are more likely to experience obstetric complications [50]. This is often due to their bodies not being fully developed for childbirth, nutritional deficiencies, and a higher risk of sexually transmitted infections [51]. Additionally, the psychosocial stress associated with the stigma of teenage pregnancy and delays in seeking medical attention [52] further contribute to these complications. A support network comprising family, friends, and partners is essential as soon as pregnancy is suspected, to link teenage mothers with youth-friendly healthcare services and to ensure that they attend medical appointments promptly, thereby preventing potential obstetric complications. Unfortunately, this support is often lacking due to strained relationships with significant individuals in their lives, as observed in our study. When providing comprehensive sexuality education to young girls, the focus should go beyond pregnancy prevention to include guidance on the appropriate steps to take in case a pregnancy occurs. Such knowledge can empower teenage girls to make timely and informed decisions if they find themselves in this situation. The advocacy strategies proposed in this study offer an avenue to inform and empower teenage girls to make informed decisions about their sexual and reproductive health.

The findings further reveal a challenging life with TPM typified with inadequate support and strained relationships. With the legal and cultural connotations of TPM, these teenagers are deserted at the moment when social support is critical. A feeling of abandonment from people who seemed to care and love them was evident in the stories of teenage mothers. The findings resonate with studies that have documented the emotional and relational challenges faced by teenage mothers, who are often left to navigate the complexities of motherhood with little to no support from their partners or families [53].

We found that the level of difficulty and regret occasioned by TPM was linked to support and acceptance the teenage mothers received. While the birth of a child may bring some joy, it is often overshadowed by the challenges of motherhood and the resentment from parents, partners, and community members. With no prior experience of childcare, no mentorship, and no source of income to meet their own and the children’s basics, teenage mothers find themselves solely in uncharted territory. The obligations of parenthood place additional demands on the stage of development of teenage girls. Although many brave this, it comes with several tradeoffs such as failure to return to school, leaving their newborns at home to look for petty jobs, and living in poor conditions.

Efforts to prevent TPM notwithstanding, pregnant teenagers and teenage mothers ought to be supported to navigate through the new challenges in their lives. The healthcare worker, who often interface with these girls when they seek maternal and childcare services should play a pivotal role to undertake restorative measures. First, to provide non-judgmental psychosocial support to the teenage girls and all the information about their health, that of their babies, as well as any services they may require. This should be provided in the context of youth-friendly services currently existing in most healthcare facilities in Uganda. Second, to act as technical advocates to negotiate a reunion with parents or partners. Third, to link teenage mothers with community-based, governmental, and non-governmental organizations that provide livelihood support to young people in vulnerable situations. The group therapy in such organizations, experience sharing, and life skills can empower these teenage girls to reclaim their esteem and gain a positive perspective to life after a pregnancy or childbirth.

### Study strengths and limitations

The strength of this study primarily lies in the phenomenological design, which enabled a deep exploration of TPM, offering nuanced insights. By focusing on personal perspectives, we uncovered meanings that are highly relevant to the participants. Moreover, the design allowed us to consider the context of participants’ experiences, enhancing our understanding of TPM within a real-life setting. However, the study also had some limitations. First, due to the interpretive nature of hermeneutic phenomenology, the researchers’ personal interpretations, informed by their prior knowledge and experience of TPM, may have influenced the findings. Second, the strong emphasis on context during data analysis and interpretation limits the transferability of our findings to other settings.

## Conclusion

The findings of this study highlight the complex nature of teenage pregnancy and motherhood. The experiences of the teenage girls were characterized by surprise, fear, ambivalence, and a profound sense of inadequacy in the support they received. These challenges were compounded by strained relationships and a diminished sense of social value, underscoring the need for comprehensive strategies that address not only the physical health of teenage mothers but also their emotional and social well-being. Improving support systems and addressing the socio-cultural factors that contribute to the stigmatization of teenage mothers are crucial steps towards better outcomes for these young women and their children. Future studies could focus on interventions to reduce stigmatization, strengthen community-based support systems, and promote positive relationships for the wellbeing of both teenage mothers and their children.

## Supporting information

S1 File Data set. (PDF)

## References

[1] United Nations Population Fund (UNFPA). State of the World’s Population 2022: Seeing the Unseen – The case for action in the neglected crisis of unintended pregnancy. United Nations Population Fund. 2022. Available from:https://www.unfpa.org/resources/state-worlds-population-2022-seeing-unseen

[2] Loaiza E, Liang M. Adolescent pregnancy: a review of the evidence. Unfpa; 2013.

[3] UNICEF. Adolescent pregnancy: A review of the evidence. UNICEF. 2021. Available from: https://www.unicef.org/reports/adolescent-pregnancy-review-evidence

[4] Anena MR, Orishaba J, Mwesigwa D. Literature review of teenage pregnancy in Uganda. 2020.

[5] Tebb KP, Brindis CD. Understanding the psychological impacts of teenage pregnancy through a socio-ecological framework and life course approach. Semin Reprod Med. 2022;40(1–02):107–15. doi: 10.1055/s-0041-1741518 34991169

[6] Manzi F, Ogwang J, Akankwatsa A, Wokali OC, Obba F, Bumba A, et al. Factors associated with teenage pregnancy and its effects in Kibuku Town Council, Kibuku District, Eastern Uganda: A cross sectional study. 2018.

[7] Kakal T, Nalwadda C, van Reeuwijk M, van Veen M, Kusters L, Chatterjee O, et al. Young people’s choice and voice concerning sex and relationships: effects of the multicomponent Get Up Speak Out! Programme in Iganga, Uganda. BMC Public Health. 2022;22(1):1603. doi: 10.1186/s12889-022-13919-x 35999598 PMC9396562

[8] Chacko S, Kipp W, Laing L, Kabagambe G. Knowledge of and perceptions about sexually transmitted diseases and pregnancy: a qualitative study among adolescent students in Uganda. J Health Popul Nutr. 2007;25(3):319–27. 18330065 PMC2754036

[9] Namuwonge F, Kizito S, Ssentumbwe V, Kabarambi A, Magorokosho NK, Nabunya P, et al. Peer pressure and risk-taking behaviors among adolescent girls in a region impacted by HIV/AIDS in Southwestern Uganda. J Adolesc Health. 2024;74(1):130–9. doi: 10.1016/j.jadohealth.2023.08.006 37804302 PMC10841615

[10] Liang M, Simelane S, Fortuny Fillo G, Chalasani S, Weny K, Salazar Canelos P, et al. The state of adolescent sexual and reproductive health. J Adolesc Health. 2019;65(6S):S3–15. doi: 10.1016/j.jadohealth.2019.09.015 31761002

[11] Ruzibiza Y. ’They are a shame to the community … ’ stigma, school attendance, solitude and resilience among pregnant teenagers and teenage mothers in Mahama refugee camp, Rwanda. Glob Public Health. 2021;16(5):763–74. doi: 10.1080/17441692.2020.1751230 32264792

[12] Råssjö E-B, Kiwanuka R. Views on social and cultural influence on sexuality and sexual health in groups of Ugandan adolescents. Sex Reprod Healthc. 2010;1(4):157–62. doi: 10.1016/j.srhc.2010.08.003 21122615

[13] Ninsiima AB, Leye E, Michielsen K, Kemigisha E, Nyakato VN, Coene G. “Girls Have More Challenges; They Need to Be Locked Up”: a qualitative study of gender norms and the sexuality of young adolescents in Uganda. Int J Environ Res Public Health. 2018;15(2):193. doi: 10.3390/ijerph15020193 29364192 PMC5858264

[14] Ninsiima AB, Michielsen K, Kemigisha E, Nyakato VN, Leye E, Coene G. Poverty, gender and reproductive justice. A qualitative study among adolescent girls in Western Uganda. Cult Health Sex. 2020;22(sup1):65–79. doi: 10.1080/13691058.2019.1660406 32045321

[15] Mangeli M, Rayyani M, Cheraghi MA, Tirgari B. Exploring the challenges of adolescent mothers from their life experiences in the transition to motherhood: a qualitative study. J Family Reprod Health. 2017;11(3):165–73. 30018654 PMC6045691

[16] Abdulaziz T, Atuyambe L, Tuhebwe D, Muhumuza C. Factors associated with accessibility to teenage friendly sexual and reproductive health services in Lira District, Uganda: a case control study. 2017.

[17] Christofides NJ, Jewkes RK, Dunkle KL, McCarty F, Jama Shai N, Nduna M, et al. Risk factors for unplanned and unwanted teenage pregnancies occurring over two years of follow-up among a cohort of young South African women. Glob Health Action. 2014;7:23719. doi: 10.3402/gha.v7.23719 25150027 PMC4141943

[18] Shaikh S, Shaikh A, Shaikh S, Isran B. Frequency of Obstructed Labor in Teenage Pregnancy. Nepal J Obstet Gynaecol. 2013;7(1):37–40. doi: 10.3126/njog.v7i1.8834

[19] World Health Organization. Adolescent pregnancy. World Health Organization. 2022. Available from:https://www.who.int/news-room/fact-sheets/detail/adolescent-pregnancy

[20] Eliner Y, Gulersen M, Kasar A, Lenchner E, Grünebaum A, Chervenak FA, et al. Maternal and neonatal complications in teen pregnancies: a comprehensive study of 661,062 patients. J Adolesc Health. 2022;70(6):922–7. doi: 10.1016/j.jadohealth.2021.12.014 35165030

[21] Ochen AM, Chi PC, Lawoko S. Predictors of teenage pregnancy among girls aged 13-19 years in Uganda: a community based case-control study. BMC Pregnancy Childbirth. 2019;19(1):211. doi: 10.1186/s12884-019-2347-y 31234816 PMC6591948

[22] Barcelos CA, Gubrium AC. Reproducing stories: strategic narratives of teen pregnancy and motherhood. Soc Probl. 2014;61(3):466–81. doi: 10.1525/sp.2014.12241

[23] Duby Z, McClinton Appollis T, Jonas K, Maruping K, Dietrich J, LoVette A, et al. “As a Young Pregnant Girl… The Challenges You Face”: exploring the intersection between mental health and sexual and reproductive health amongst adolescent girls and young women in South Africa. AIDS Behav. 2021;25(2):344–53. doi: 10.1007/s10461-020-02974-3 32683636 PMC7368608

[24] Otegbayo BE, Omar N, Danaee M, Mohajer S, Aghamohamadi N. Impact of individual and environmental factors on academic performance of pregnant adolescent. BMC Womens Health. 2023;23(1):383. doi: 10.1186/s12905-023-02520-y 37480050 PMC10362692

[25] Essama-Nssah B, Leite PG, Simler KR. Achieving universal primary and secondary education in Uganda: Access and equity considerations. Washington, DC: World Bank, Poverty Reduction and Equity Group; 2008.

[26] Moses KM, Liu WT. The role of TVET skill development in transformation of informal sector in developing countries: the case study of skilling Uganda program in Kampala urban area Uganda. In: Proceedings. Vol. 83. 2023. pp. 46.

[27] Nabugoomu J, Seruwagi GK, Hanning R. What can be done to reduce the prevalence of teen pregnancy in rural Eastern Uganda?: multi-stakeholder perceptions. Reprod Health. 2020;17(1):134. doi: 10.1186/s12978-020-00984-x 32867811 PMC7457815

[28] Ninsiima LR, Chiumia IK, Ndejjo R. Factors influencing access to and utilisation of youth-friendly sexual and reproductive health services in sub-Saharan Africa: a systematic review. Reprod Health. 2021;18(1):135. doi: 10.1186/s12978-021-01183-y 34176511 PMC8237506

[29] Renzaho AMN, Kamara JK, Georgeou N, Kamanga G. Sexual, reproductive health needs, and rights of young people in slum areas of Kampala, Uganda: a cross sectional study. PLoS One. 2017;12(1):e0169721. doi: 10.1371/journal.pone.0169721 28107371 PMC5249247

[30] Wood L, Hendricks F. A participatory action research approach to developing youth-friendly strategies for the prevention of teenage pregnancy. Educ Action Res. 2016;25(1):103–18. doi: 10.1080/09650792.2016.1169198

[31] Sachs JD. From millennium development goals to sustainable development goals. Lancet. 2012;379(9832):2206–11. doi: 10.1016/S0140-6736(12)60685-0 22682467

[32] Zahavi D. The practice of phenomenology: the case of Max van Manen. Nurs Philos. 2020;21(2):e12276. doi: 10.1111/nup.12276 31441216

[33] Wang C, Burris MA. Photovoice: concept, methodology, and use for participatory needs assessment. Health Educ Behav. 1997;24(3):369–87. doi: 10.1177/109019819702400309 9158980

[34] Kimera E, Vindevogel S. Photovoicing empowerment and social change for youth living with HIV/AIDS in Uganda. Qual Health Res. 2022;32(12):1907–14. doi: 10.1177/10497323221123022 35998362

[35] Wang CC, Redwood-Jones YA. Photovoice ethics: perspectives from Flint Photovoice. Health Educ Behav. 2001;28(5):560–72. doi: 10.1177/109019810102800504 11575686

[36] Lindseth A, Norberg A. A phenomenological hermeneutical method for researching lived experience. Scand J Caring Sci. 2004;18(2):145–53. doi: 10.1111/j.1471-6712.2004.00258.x 15147477

[37] Ricoeur P. Interpretation theory: Discourse and the surplus of meaning. TCU Press; 1976.

[38] Van Manen M. Researching lived experience: Human science for an action sensitive pedagogy. Routledge; 2016.

[39] Laverty SM. Hermeneutic phenomenology and phenomenology: a comparison of historical and methodological considerations. Int J Qual Methods. 2003;2(3):21–35. doi: 10.1177/160940690300200303

[40] Gyesaw NYK, Ankomah A. Experiences of pregnancy and motherhood among teenage mothers in a suburb of Accra, Ghana: a qualitative study. Int J Womens Health. 2013;5:773–80. doi: 10.2147/IJWH.S51528 24250233 PMC3829679

[41] Seamark CJ, Lings P. Positive experiences of teenage motherhood: a qualitative study. Br J Gen Pract. 2004;54(508):813–8. 15527606 PMC1324913

[42] Bhandari SD, Joshi S. Perception and perceived experiences about prevention and consequences of teenage pregnancy and childbirth among teenage mothers: a qualitative study. J Adv Acad Res. 2017;3(1):164–72. doi: 10.3126/jaar.v3i1.16625

[43] Tetteh J, Nuertey BD, Dwomoh D, Udofia EA, Mohammed S, Adjei-Mensah E, et al. Teenage pregnancy and experience of physical violence among women aged 15-19 years in five African countries: analysis of complex survey data. PLoS One. 2020;15(10):e0241348. doi: 10.1371/journal.pone.0241348 33108400 PMC7591093

[44] Chambers BD, Erausquin JT. The promise of intersectional stigma to understand the complexities of adolescent pregnancy and motherhood. J Child Adolesc Behav. 2015;3(4):249.

[45] UNICEF. Ending child marriage and teenage pregnancy in Uganda. 2015. [cited 24 Aug 2024]. Available from: https://www.unicef.org/uganda/media/3901/file/Formative%20Research%20Ending%20Child%20Marriage%20and%20Teenage%20Pregnancy%20in%20Uganda.pdf

[46] Parikh SA. “They arrested me for loving a schoolgirl”: ethnography, HIV, and a feminist assessment of the age of consent law as a gender-based structural intervention in Uganda. Soc Sci Med. 2012;74(11):1774–82. doi: 10.1016/j.socscimed.2011.06.037 21824700 PMC3265666

[47] Namasivayam A, Schluter PJ, Namutamba S, Lovell S. Understanding the contextual and cultural influences on women’s modern contraceptive use in East Uganda: A qualitative study. PLOS Glob Public Health. 2022;2(8):e0000545. doi: 10.1371/journal.pgph.0000545 36962757 PMC10022157

[48] UNAIDS. Global HIV & AIDS Statistics – Fact Sheet. UNAIDS. 2021. [cited 24 Aug 2024]. Available from:https://www.unaids.org/en/resources/fact-sheet

[49] Fotso JC, Cleland JG, Muki B, Adje Olaïtan E, Ngo Mayack J. Teenage pregnancy and timing of first marriage in Cameroon-What has changed over the last three decades, and what are the implications?. PLoS One. 2022;17(11):e0271153. doi: 10.1371/journal.pone.0271153 36395149 PMC9671313

[50] World Health Organization (WHO). Adolescent Pregnancy. WHO. 2018. [cited 24 Aug 2024]. Available from:https://www.who.int/news-room/fact-sheets/detail/adolescent-pregnancy

[51] Treffers PE, Olukoya AA, Ferguson BJ, Liljestrand J. Care for adolescent pregnancy and childbirth. Int J Gynaecol Obstet. 2001;75(2):111–21. doi: 10.1016/s0020-7292(01)00368-x 11684107

[52] Mkhwanazi N. Understanding teenage pregnancy in a post-apartheid South African township. Cult Health Sex. 2010;12(4):347–58. doi: 10.1080/13691050903491779 20162476

[53] Meade CS, Ickovics JR. Systematic review of sexual risk among pregnant and mothering teens in the USA: pregnancy as an opportunity for integrated prevention of STD and repeat pregnancy. Soc Sci Med. 2005;60(4):661–78. doi: 10.1016/j.socscimed.2004.06.015 15571886

