# Supplementary material for: Photo-voicing experiences of teenage mothers with teenage pregnancy and motherhood in Western Uganda
Source: PLoS One. 2025 Nov 13;20(11):e0335413. doi: 10.1371/journal.pone.0335413 (PMC12614584; doi:10.1371/journal.pone.0335413)
Supplement: S1 File — (PDF) [file pone.0335413.s001.pdf]

Group interview of adolescent mothers

Introduction

My name is (NAME OF A PERSON) my village is (name of a place) I am not married I have one child, I am 18 years old and both my parents are alive.

My name is (NAME OF A PERSON) 18 years from (name of a place), I am married with one child.

My name is (**(NAME OF A PERSON)**), from (name of a place) I am 17 years, am not married with one child all my parents are alive.

My name is (NAME OF A PERSON) I am 19 years I am married from (name of a place) I have one child.

My name is (NAME OF A PERSON) 18 years I have one child and i am from (name of a place) village both my parents are alive.

My name is (NAME OF A PERSON) i am from (name of a place) i am 18 years I have two children but i am not married.

My name is (NAME OF A PERSON) from (name of a place) I have one child i am married both my parents are alive.

My name is (NAME OF A PERSON) from (name of a place) 17 years i am married and both my parents are alive I have one child.

My name is (NAME OF A PERSON) I have one child both my parents are alive I have one child; I have come from (name of a place).

Me (NAME OF A PERSON) I studied up to S.3 that is when I got pregnant. When I told my boyfriend he accepted he didn't refuse but he told me what should we do? Because we are still at school. He told me should we tell the parents? I tell my parents and he tells his parents. I first told him it is not possible; he asked me why. I told him we cannot say that now because it will bring problems. We looked for what to do. I first left home and went to (name of a place) to my sisters because it was where they wanted me to go and study from for some time. So, when I looked for what do and failed, I decided to go to (name of a place) so that they comfort me. When I reached there, after two weeks my sisters started seeing my life was not as usual, I had changed and looked like a pregnant woman. They [sisters] first feared to ask me because

they [sisters] knew I was not easy. They [sisters] went to work and talked about it. When they [sisters] came back with my eldest brother asked me that are you pregnant? Thats because you have all signs of pregnancy. I told him [brother] yes aim pregnant, they asked me whose pregnancy is it, I told him the boy, they asked me where the boy stays, I explained to him. Then they said that for us we have known that you are pregnant but your parents do not know what should we do? You tell us, I didn't have words I was crying. Then my brothers said we are leaving because now you have anxious, you are not talking, you are just crying. When they came back they said we have to call our parents and tell them and you have to go back to (name of a place) and look for that boy if not when the parents learn about it you and the boy will be imprisoned and we as your sisters and brothers we may not be able to support you. We first called mummy at home she is the first one I told not Dad and they explained to her. Like a parent, she first got annoyed then later she calmed down. She asked them to let me go back home, they said no Daddy is a problem. They told her if we send this girl we know daddy will imprison both of them and that is not the solution. The solution is to call the boy and ask him if he can look after the girl. When they called the boy he was not bad, he accepted that the pregnancy as his and he said that I will look after the girl. So, that time I left (name of a place) and came to the boy's home and even at the market that is where aim.

How did your daddy know?

Mum is the one who told Daddy, he got annoyed but said one thing, let my daughter come back home even now he calls me to go back and start school. I am preparing to go back home.

How did the community perceive it?

You know like rumours among people not everybody wishes you well these who were happy were happy those who felt pain felt pain.

Did you attend ANC?

Yes, I did ANC from (NAME OF HOSPITAL) when I came back from (name of a place) in October I started ANC.

(NAME OF A PERSON)

I had gone to work in (name of a place) I got a man and he married me, I got the first pregnancy which ended up into abortion. I got the second pregnancy but I did not know that I was pregnant, when it reached 5 months I got misunderstanding with the man and I came back

home. At 6 months the pregnancy wanted to come out. I went to (NAME OF HOSPITAL), they treated me, I took medicine when it reached eight months it again wanted to come out, they told me to pray had, I started praying until I delivered, my child birth process was hard.

How did the parents know you were pregnant?

I don't stay with my mother my father got another woman, so when I came back I went and started staying with my grandmother. When time for delivery reached, I went to hospital after delivering they told me that my uterus is tone that they were going to operate me and repair it then take it back but God helped me they found I was okay. The man is there but he does not give me any help, he is not looking after the baby and even when I went to hospital he did not support me. I got a job after delivering at (name of a place) to work in a hotel but fell sick because I started working early after birth at 2 months so they told me to first leave work and first be well.

(NAME OF A PERSON)

I got pregnant when I was working as a maid, when I went home my had left home and even up to now she does not know that I have a child, she separated with my father even now I don't know where she is.

How did you know you were pregnant?

I had spent 2 months without seeing my periods that is when I know I was pregnant then I started attending antenatal at (NAME OF HOSPITAL). When I told the man that I was pregnant, he refused that it was not his pregnancy, I stayed with my pregnancy without any help from him, when labour started I went to (name of health facility) HCIV, I called him and told him they have asked for money I request for money he said I will not send you money because that pregnancy is not mine and that baby is not mine.

They discharged me home I looked after my child; I developed puerperal sepsis and I convulsed I was at my grandmother's home. my grandmother said she cannot manage me I went back to boss at work; she looked after me and gave me everything up to now the man has never given me any help I am the one looking after my baby.

Where did you got money to care for your baby?

I stay with my sister she does not have a job but is just married. My dad told me I had gone to work I should take for him money not the child. So, I decided to keep with my sister.

(NAME OF A PERSON)

Me I got pregnant I had not understood it, I was still schooling in P.7 I got a man from our place, then we came this way to work. When I saw I had reached in May without going into my menstruation after having sex with him in April, I just knew I was pregnant. I feared to tell my parents. I told my boyfriend that it seems I am pregnant. The boy asked me how do you know it? I answered him that me I have my way I have known it. At first, he did not accept, then I told him should I abort it, he said no don't try to abort it. Then I asked him will you be able to look after me, he said yes. By that time, I was at home and my parents were not aware. I told my parents that I was going to work, in town they refused then I ran away from them.

When I told my boyfriend that I had ran away from home he told me to go and stay with him in (name of a place), he looked after me, I started going for antenatal at (name of health facility) HCIV and even delivered from there but my Mum did not know she knew it when I was 6 months pregnant. She kept asking me to go back home but I was fearing there, I knew she was going to beat me. At 6 months the pregnancy wanted to come out, I went to (name of health facility) HCIV they treated me and I became fine. I went home and I stayed there for 3 months then came back to my husband.

Did you go home when you were still pregnant or after birth.

I went there after giving birth, my parents saw the baby and became happy.

When I went back to my husband for like one week, he started mistreating me, I have first left his home, I am now staying with my sister in (name of a place). Now he is calling me that I should go back to him now, I don't know what I can do.

How was he mistreating you?

He started showing me things I don't understand, there is a girl who came and started telling me that this home was mine. When I asked my husband he refused. Now I am at home he is calling me to go back but I have refused.

(NAME OF A PERSON)

Me I understand that I was pregnant when it was 6 months old, it was very had to tell my parents, my boyfriend did not force me to have sex we all agreed, were studying together. After getting pregnant I first told my sister who stays outside the country, I called her and told her but she told me to tell my parents. It was hard for me I first went to my other sister in (name of

a place) I stayed there then there she said let’s go home, we came back home two of us we told our mother. Our mother asked me about the boy, I told her, she went to the home of a boy he was not there, he is a soldier. His parents called him and he came the parents were angry. When he came, he was told and he accepted to take care of me. I stayed with my mother but she told me after giving birth when the baby starts walking you will go back to school and finish your studies. That’s how it was up to now I waiting for the baby to begin walking so that I go back to school.

Now what is your husband saying.

My husband is not saying anything but he is taking care of his child.

How did people around you react on it.

Some people were not happy about it those who don’t wish me well were happy.

Where did you deliver from and how were you treated?

I delivered from (NAME OF HOSPITAL), they handled me well they were something missing which needed money my mother-in-law, she is the one who paid.

(NAME OF A PERSON)

Me I was schooling I was finishing P.7 I first looked for the job, when I got a job I started working as a shopkeeper, my boss is the one who impregnated me at 14 years. the first pregnancy, he forced me to have sex with him. After forcing me to have sex, I did not know that he had impregnated me, he told me he was taking me to (name of a place) to work in his other businesses. When I reached there, I found out I was pregnant.

How did you know you were pregnant and what happened.

I finished 2 weeks while spitting all the time having dizziness, I went and bought HCG strip and tested my urine. I found out I was pregnant, I did not tell my parents I had many thoughts I asked myself how I will tell my parents I told the man that I am pregnant, he kept quiet for one month without talking to me. After one month I called him and told him I am going back home, he said no don’t go back home. Be there I will look after you in (name of a place). I stayed there when the pregnancy made 6 months, he stopped sending me help I left (name of a place) and came back. this was with little money I had worked I rented at (name of a place). I stayed there when time for delivery reached that is when I called my parents while going to the hospital. I told them I got pregnant and right now I am in (NAME OF HOSPITAL) and I am

going to give birth. They did not replay me after delivery the man cared for me, he took me at their place, I stayed there when the baby was 1½ years I got another pregnancy when I was not ready.

When I told him he said that second pregnancy is not mine, it might be for other men, he became angry a lot, I went back home and told my parents to arrest him. My aunt called me and said first come and we stay together so that we first talk with that man. Even when we talk with him, there is nothing he is doing for me.

Now where is the other baby

Iam staying with them all at my aunts' place. The man is looking after the first baby not this one. He completely refused the second child.

How was the reaction of the people around home?

Others got annoyed others went around talking that I got an elderly man that I should leave him.

When you went to deliver in the hospital who paid the bills.

My aunt paid the hospital bills; my aunt is the one even looking after this second child.

(NAME OF A PERSON)

Me when I got pregnant my parents had failed to raise my school fees and I was in P.6. I don't stay with my parents I stay with my untie in (name of a place). I started working as a maid in (name of a place). that's when I got pregnant. I knew I was pregnant when I completed one month without seeing my periods started feeling feverish, lost appetite. The man did not force me, we agreed to have sex when I told him that I was pregnant he didn't refuse, he told me lets first go to the hospital. I told my boss everything and asked her permission to go to the hospital when we reached the hospital, they tested me and found out I was pregnant. I came back and told my boss; she told me we have to call your aunt, so she called my aunt and my aunt told me to tell the man to send her money so that she comes and meet him and they talk. The man sent the money to my aunt. They met and talked but I don't know what they discussed. My aunt was not happy but she had nothing to do, my aunt first feared to tell my Daddy. she told my Daddy when I was almost delivering. The man cared for me even at the time of giving birth he supported me.

Where did you deliver from?

I first did my antenatal care from here at (NAME OF HOSPITAL) then later went home my boss told me to go home not to give birth from her home. But I stayed with my boss I went home when I was remaining with two months to give birth. At home I attended ANC twice then gave birth.

How did your daddy know and what wan his reaction

My aunt told my Daddy when I was left with only one month to give birth

My daddy got annoyed and said he will come to see the man but up to now he has not come because he stays in (name of a place) he does not stay with my mum.

The man is caring for us even now the baby is sick I have taken him to the man and he is treating the baby. Is there any problem you are facing? No major problem only that my baby is sick, and also when I was pregnant it had entered badly, I went to hospital and the nurse told me the baby was in breech presentation the nurse told time will come and baby will turn to cephalic. When I went home they gave herbal medicines which helped me to deliver well. I were preparing to operate me but God helped me and I was not operated.

What was the reaction of the community?

Others were happy others not happy and others started rumour mongering.

How was it at the hospital?

At the hospital I found few beds they told me to be walking outside so the strong contraction found me outside and I delivered from outside. They called the nurse to help me, who refuse so they gave her bribe, that is when she came and cared for me. I bled to much but they managed me. At the hospital, I was with my aunt and a neighbour.

(NAME OF A PERSON)

Me I had stopped schooling in P.3 I had gone to work as a maid in (name of a place), that when I got a man we talked well we talked for a long time before getting into love then he started talking about love but still spent 6 months without having sex the I went then I went to (name of a place) to work, I called me to come back and stay with him. I came back and stayed with him for 5 months so I got pregnant, I told my parents, they had no problem with me because I was not at school.

How did you know you were pregnant

I was prepared to get pregnant; I wanted to conceive so that I deliver my baby when I conceive, I was happy and my parents were happy. Am staying with my husband up to now though his income is not enough but his taking care of us. I don't have any problem with taking care of the baby.

(NAME OF A PERSON)

Me in the hospital I did not handle me well. When I reached there at 5:00am they asked me here if you lose blood we don't have boda boda to take you to (NAME OF HOSPITAL), do have money to buy blood?

I told them I don't have money and I staying here I am not going to (name of hospital) because everything there is money. I was at (name of facility) HCIV

At that time did you have no blood in your body.

No, she told me if I deliver and bleed too much, we have no blood here. The nurse did everything possible she asked me my name and I told her my name is (name of a person), she told me if I don't call Jesus, she will not work on me well. I told her I will keep calling my Owobusobozi (Local word for God) but I will not call Jesus, she helped me and I gave birth. After delivering the baby the placenta retained and baby failed to cry, baby was born with 4.6kg they bagged the baby injected the baby with drugs but refused to cry. The placenta had failed to come out the nurse put her hands inside my vagina and kept saying Jesus and for me kept saying Owobusobozi (Local word for God). She put her finger inside to get it out it broke 3 times me I kept calling Owobusobozi (Local word for God).

She told me (name of a person) you have refused to call Jesus at birth and on placenta removal. Now I am going to suture you without giving you anaesthesia and I see if you will not call Jesus. I told her even if you suture me without that drug, I will not call Jesus.

She suturing me I would feel her injecting and pulling and felt a lot of pain I cried to much I called my attendant to come and help me she refused and ran away crying. But nurse continued to suture me until she completed.

The nurse now told me that have seed what you have done to refuse calling Jesus, I told her there is no problem the good thing you have finished.

(NAME OF A PERSON)

Me I got Labor pains at around 6pm it became worse at 2:00am we went to the hospital there care was there but there was a cleaner who was disturbing us. She made us carry boxes they could not understand whether you have pain or no, some of us sutures were paining us she gave a condition to carry the boxes in order to go on bed and sleep

What was in boxes?

They were like these patients' cupboard she was cleaning she first put all outside then after cleaning she told every mother to carry one each and take them inside. Nurses handled us well

(NAME OF A PERSON)

Me that health workers handled me badly when I was 3 months pregnant, I got malaria. When I was going to the hospital I convulsed on the way, on reaching on the ward they told me to come back at OPD so that the test me for blood and urine and ultra sound scan and I was badly off. After testing they told me that the malaria was too much go back up and they treat you that time I didn't have energy of walking I was crawling.

When I reached on ward, they denied me a bed and told me to sit on a bench, I until I collapsed down that is when they gave me a bed, those I had come with are the ones that carried me onto the bed.

On giving birth, I left home and went to the hospital I found the nurses, they told them I am badly off but they said I first sit down and wait one nurse came and examined me and said the baby is coming but first keep on the bed. I stayed on the bed I, cried but there was no tears, the nurse came back we talked to her, she asked for the money, we gave her then she delivered me.

(NAME OF A PERSON)

Me I started feeling Labor pains at 3:00pm contractions were frequent and strong, I told my aunt lets go to the hospital she told me to patient until it is 8:00pm so that we go when people are not seeing us. I told her the pain is much let's go, my aunt prepared and my other sister we sent.

At 5:00pm almost 6:00pm I gave birth after delivering they said my uterus was torn we go and buy drugs and pay money for operation so that I am operated. My aunt went to buy the drugs remained on bed praying so that they don't operate me.

God helped me when I reached in the uterus, I was bleeding too much. In theatre they examined me and put instruments inside me, by God's grace they found mu uterus was intact. But all

what we had bought remained with them things like drugs gauze, gloves soap in the bucket the total money was spend was 180,000/=

This money (180,000/=) I got it while digging in peoples shambas when I was pregnant. I used to dig they pay me and save money.

(NAME OF A PERSON)

Me at (name of hospital) the nurse told me free things got finished they no longer bring for us drugs there only things for buying for us here we write for you and you go to buy the drugs.

(NAME OF A PERSON)

I first went to (name of health facility) I told the nurse that my pregnancies are always ending in abortion, she wrote for me and told me to go and do scan. When I went to (name of hospital) I found a woman who was short and brown she was talking Rukonjo, I told her that they have sent me here you do me a scan. She told me do you have 35,000/=. I told her I don't have she told me to sit there If I get that money she will do it for me.

I thought may be the woman was annoyed for other things she will call be later. After sometime she came back and saw are and bypassed me. I asked her you woman they have sent me here to do scan and they told me it is for free she said why do you like free things free things are not here they got finished. If you want them go to town and become a beggar.

What pained me was another woman came speaking (language) she didn't have money but she told her to enter and worked on her. Me I stayed there the whole day what I did I left and went back home and promised not to go back to (name of hospital). Now where did you deliver from?

At (name of health facility) it is where I did my antenatal from and even delivered from there. The nurse there cared for me.

(NAME OF A PERSON)

Just in (NAME OF HOSPITAL) they got 20,000/= for anaesthesia when they were going to suture me then doctor came and also took 50,000/= because I had a lot of pain and gave me painkillers.

Iam happy because God gave me alive child there is no disease disturbing her man loves his child, he carries the baby and plays with the baby. Some men don't like their children but my husband loves our child I also love my child when the baby smiles for I become happy.

Disadvantages of getting pregnant and delivering when still young/challenges

When I gave birth to my child, the baby started crying stop crying. I thought the baby had abdominal pain. When I touched the abdomen, it was hard got worried and shaved my grandmother.

She said that is how the baby's body is I asked her does the baby's body hard like these, I told her to touch and she touched and said Mawee we are killing the baby. We took the baby to (NAME OF HOSPITAL), the baby was put on oxygen therapy and put NG tube where we were feeding it with milk. We spent there on week we bought the drug for 15,000/= 3 days after discharge the baby got cough I didn't take her to hospital, I had started working I went and bought drugs I bought two syrups for 10,000/=

(NAME OF A PERSON)

Me what is worrying me my baby is not breastfeeding well and not eating well crying.

(NAME OF A PERSON)

My baby is always getting bowls on the body. I took him in the hospital and wrote for me drugs but even when I gave him the drugs the no improvement. The wounds had attached the baby's head and week.

(NAME OF A PERSON)

I thank God that my baby is growing but when he gets a cough the baby can cough and vomit then ask myself why did I deliver this baby/ The father is not giving me help I ask myself a lot of things and worry so much, I ask myself a lot of questions. Then later normalize and leave things say I am the one who gave birth to this child, let me settle and look after my baby.

(NAME OF A PERSON)

My baby developed cough and flu we went to fort pharmacy and explained to them that we have a baby who is 2 months and 3 weeks and has too much cough and flue, when on sneezing there is blood coming from the nose the drug was not working. We were advised to take the baby to the pediatrician at (NAME OF HOSPITAL).

(NAME OF A PERSON)

We don't have money to buy clothes for our children. The clothes we bought for newborn have now not fitting the babies now I am asking myself where I get money to buy clothes.

Me my child I started her on the cow's milk I work in town and some from (name of a place) and baby is very heavy. Baby is taking a lot of milk and I am failing to buy milk when I get money I buy one cup. I leave the baby with my grandmother.

Me when I take my baby at our home, she falls sick but when we come back to the father the baby is fine.

My baby is always getting skin rashes on her body/

(NAME OF A PERSON)

When you give birth there are a lot of things you learn like with bad experience I didn't admire to give birth again you even fear to get another man thinking that he might be mistreat you like the other one.

At home they don't see you as an important person. Because you have given birth from home your children there disturbing them.

They begin telling you to contribute at home because you have children there. You here your parents saying now you also buy soap, sugar, salt smearing oil.

Some of us stay with our sisters some times our sisters leave everything on our heads like buying things to use at home. Remember we are not working and my husband the phone is off. So you have to look around and get something some time we stay hungry. Even they reach an extend of hiding things like food just because you have not contributed.

What should we do to make men be responsible for their children?

(NAME OF A PERSON)

I think I should get money board a car and go to (name of a place), maybe he can help me.

(NAME OF A PERSON)

Like me the man forced me to have sex with him and another thing he is very old, but has even failed to look after his children what should I do.

I talked with him in peace, he refused and decided to switch off his phone I called on his brother's phone but hearing my voice he disconnected the call. My in laws have not helped me either.

How can we help those others who are young at school

Those who have a chance to study they should study and don't go into giving birth (Kobugabe Jane)

Those who are not yet pregnant let them protect themselves even if you have a boy make sure, he does not impregnant you (Kemirembe).

Others girls even if you tell them they will not accept want to first get into the problem.

We should tell them that in marriages there is nothing good only suffering you have a chance to study first study.

We sit with them and tell them what is good and what is bad and let them decide on their own. We were told but decided not to listen so here we are.

It is not good to advise a young girl to use modern family planning methods. You will be destroying her body. This may make her infertile forever; some develop fibroids in the uterus ((NAME OF A PERSON)) the nurses told us that if you take long time without seeing menses that blood gets stuck inside and you develop fibroids.

What I know family planning methods don't treat us the same there those that menstruate normally and others don't menstruate at all. We should not stop our fellow girls from using them.

We who have experienced this we should counsel these young girls and help them out. To always abstain from sex.

Those who have boyfriends we should counsel them to start on a modern FP method.

I continued with school but I reached time and found that the uniform was not fitting me. I was small and I had two uniforms now they were not fitting me and I was fencing to tell parents.

When we got holidays, he said the way i was not going to study coaching I should tell people at home to first send me my relatives in (name of a place).

My parents were not bad when I told them they prepared me well, they even gave me my younger sister who was 4 years and told us go and spend you holidays there. For me what took me there was the pregnancy, I had refused to (name of a place) but I knew if didn't go they were going to arrest my mother in law and the boy.

When I reached there after 2 weeks my sisters so my life had changed, I was vomiting and sickly all the time. They were seeing my eyes were very white. They feared to ask me because they knew me, I may answer them badly they went to work when they came back, they asked me are you pregnant/

I first hid from them and told them am not pregnant.

They sent my brother my brother came and asked me are you pregnant I answered him yes am pregnant.

My brother went back and told them that for me she has told me she is pregnant.

We sat one of my sisters said you know how our father is hot and our mother is hot what should we do me I kept quiet.

They said now we are going to sit and call your husband and he listens to what we are going to say because when we go back to (name of a place) your man will go to jail.

My brother said all that is useless the girl has been impregnated after giving birth she will go back and other than arresting her or the boy which is not a solution

We sat as children before telling parents to get a solution. They asked me will the boy take care of you. I said he has no problem because even I told him I am pregnant he didn't refuse he only asked me what we can do.

At that time, I had no words, I had nothing to do I was crying all the time.

My sisters told me if the boy has said he is going to take care of you what should we tell our parents.

They told me they had nothing to do you are going to call your mother and tell her how you are pregnant and ask her for forgiveness, before your Daddy know it. Your mother will be the one to tell your father and will get ways of calling him.

So, we sat as children and discussed how to tell our mother we called Mummy right and we talked Mummy said why can't you send her and comes back this way. The older sister refused

and said if she comes back their dad will arrest her and the boy and there will be no solution. The boy has accepted to give her help if they arrest him, he will stop working.

At that time, we looked for what to do after telling Mummy. So here Mummy told me this is what you have pain me with all the money I have put in you, we have been giving you everything you needed so these are the benefit you have brought for us..

Me I had not talked to Mummy. I was fearing it was my sister who talked to her. I looked for where to start from and I failed.

My Mummy said you know you father how tough he is how are we going to tell him.

I stayed in (name of a place) for a long time then Dad called asking if am not coming back to school but I was supposed to start school from (name of a place) my sister told my Dad that we are going to send her back to (name of a place) let her first stay here.

My mother was coming to (name of a place) they gave her my young sister I had gone with to bring her back.

Dad called again and asked me when are you coming back, schools are opening o Monday. I told him I will come on Saturday and it was on Friday.

There Mummy told him everything and he kept quiet, after sometime he said I should be sent back home. Let her come back home and I see what I can do. I refused and told them I have no where I am going because I know how my Dad is.

Every day Dad is calling me, calling my sisters that I should go back home and start school.

Up to now I have not yet gone back home because I know my dad is still angry with me.

As days went by, I told my sisters let me go back to (name of a place) and I stay with my boyfriend. They said what are you doing, if Dad learns that you are in (name of a place) you will land into problems.

Was the boy still studying?

No, the boy was working was repairing cars, he said I will take care of the girl and I will give her everything she wants.

The girls said it is okay we don’t have any problem; the boy had sent money for helping me and every week he would send money.

So, I told them let me go to my boyfriend, but my Dady would always call and tell my sisters to me back home so that I go back to school.

I came to my boyfriend in (name of a place) I came in September.

Where was his home?

In (name of a place) but when I came, we rented a house in (name of a place) that where we first stayed, we hid ourselves deep in (name of a place) there was banana plantation that's where we hid.

Experiences with TPM

Codes

Name	Description	Files	References
Being surprised and attempting to conceal the pregnancy		14	76
Wondering what to do		9	11
Being confused and surprised		13	13
Getting first signs of pregnancy		8	12
Running away from home		7	11
Fearing imprisonment for partner		5	6
Revealing pregnancy to relatives first		4	5
Fearing parental reaction		11	18
Living with ambivalence and strained relationships		13	56
Happy to get a baby		7	7

Name	Description	Files	References
Parents being disappointed		5	8
Parents happy to see baby		4	5
Mixed reactions from community members		6	7
Mistreatment from partner		10	18
Abandonment by partner		8	11
Receiving insufficient support		13	54
Attending antenatal care		9	11
Partner's role in care and support		12	12
Not getting good care at health facility		4	7
Lacking money		11	14
Relatives paying hospital bills		4	6
Living through predicaments and diminished social value		14	92
Baby crying too much		6	13
Being under looked		8	13

Name	Description	Files	References
Baby falling sick all the time		12	16
Made to do adult roles		5	5
Experiencing abortion		3	5
Getting difficulties during delivery		12	15
Getting post-delivery complications		5	9
Experiencing near abortion		3	3
Getting much pain during labor		8	13
